# A family of conjugate gradient methods for large-scale nonlinear equations

**DOI:** 10.1186/s13660-017-1510-0

**Published:** 2017-09-22

**Authors:** Dexiang Feng, Min Sun, Xueyong Wang

**Affiliations:** 1 School of Management Sciences, Fudan University, Shanghai, China; 2 Qufu Normal University, Shandong, 276826 China; 3 School of Management, Qufu Normal University, Shandong, 276826 China

**Keywords:** 90C25, 94A08, nonlinear equations, conjugate gradient method, projection method, global convergence

## Abstract

In this paper, we present a family of conjugate gradient projection methods for solving large-scale nonlinear equations. At each iteration, it needs low storage and the subproblem can be easily solved. Compared with the existing solution methods for solving the problem, its global convergence is established without the restriction of the Lipschitz continuity on the underlying mapping. Preliminary numerical results are reported to show the efficiency of the proposed method.

## Introduction

Consider the following nonlinear equations problem of finding $x\in C$ such that

1.1 $$ F(x)=0, $$

where $F: R^{n}\rightarrow R^{n}$ is a continuous nonlinear mapping and *C* is a nonempty closed convex set of $R^{n}$. The problem finds wide applications in areas such as ballistic trajectory computation and vibration systems [1, 2], the power flow equations [3–5], economic equilibrium problem [6–8], etc.

Generally, there are two categories of solution methods for solving the problem. The first one is first-order methods including the trust region method, the Levenberg-Marquardt method and the projection method. The second one is second-order methods including the Newton method and quasi-Newton method. For the first method, Zhang et al. [9] proposed a spectral gradient method for problem (1.1) with $C=R^{n}$, and Wang et al. [3] proposed a projection method for problem (1.1). Later, Yu et al. [10] proposed a spectral gradient projection method for constrained nonlinear equations. Compared with the projection method in [3], the methods [9, 10] need the Lipschitz continuity of the underlying mapping $F(\cdot)$, but the former needs to solve a linear equation at each iteration, and its variants [11, 12] also inherit the shortcoming. Different from the above, in this paper, we consider the conjugate gradient method for solving the non-monotone problem (1.1). To this end, we briefly review the well-known conjugate gradient method for the unconstrained optimization problem

$$\min_{x\in{R}^{n}}f(x). $$

The conjugate gradient method generates the sequence of iterates recurrently by

$$x_{k+1}=x_{k}+\alpha_{k} d_{k},\quad k=0,1,2,\ldots, $$

where $x_{k}$ is the current iterate, $\alpha_{k}>0$ is the step-size determined by some line search, and $d_{k}$ is the search direction defined by

$$d_{k}= \textstyle\begin{cases}-g_{k}&\text{if } k=0,\\ -g_{k}+\beta_{k}d_{k-1}&\text{if } k\geq1, \end{cases} $$

in which $g_{k}=\nabla f(x_{k})$ and $\beta_{k}>0$ is a parameter. The famous conjugate gradient methods include the Fletcher-Reeves (FR) method, the Polak-Ribière-Polyak (PRP) method, the Liu-Storey (LS) method, the Dai-Yuan (DY) method. Recently, Sun et al. [13] and Li et al. [14] proposed two variants of PRP method which possesses the following property:

$$\vert \beta_{k} \vert \leq t\frac{ \Vert g_{k} \Vert }{ \Vert d_{k-1} \Vert },\quad\forall k\geq1, $$

where $t>0$ is a constant.

In this paper, motivated by the projection methods in [3, 11] and the conjugate gradient methods in [13, 14], we propose a new family of conjugate gradient projection methods for solving nonlinear problem (1.1). The new designed method is derivative-free as it does not need to compute the Jacobian matrix or its approximation of the underlying function (1.1). Further, the new method does not need to solve any linear equations at each iteration, thus it is suitable to solve large scale problem (1.1).

The remainder of this paper is organized as follows. Section 2 describes the new method and presents its convergence. The numerical results are reported in Section 3. Some concluding remarks are drawn in the last section.

## Algorithm and convergence analysis

Throughout this paper, we assume that the mapping $F(\cdot)$ is monotone, or more generally pseudo-monotone, on $R^{n}$ in the sense of Karamardian [15]. That is, it satisfies that

2.1 $$ \bigl\langle F(y), y-x^{*}\bigr\rangle \geq0, \quad\text{for all } y \in{R}^{n}, x^{*}\in{S}, $$

where $\langle\cdot,\cdot\rangle$ denotes the usual inner product in ${R}^{n}$. Further, we use $P_{C}(x)$ to denote the projection of point $x\in R^{n}$ onto the convex set *C*, which satisfies the following property:

2.2 $$ \bigl\Vert P_{C}(x)-P_{C}(y) \bigr\Vert ^{2}\leq \Vert x-y \Vert ^{2}- \bigl\Vert P_{C}(x)-x+y-P_{C}(y) \bigr\Vert ^{2}, \quad\forall x,y\in R^{n}. $$

Now, we describe the new conjugate gradient projection method for nonlinear constrained equations.

### Algorithm 2.1

*Step* 0.: Given an arbitrary initial point $x_{0}\in R^{n}$, parameters $0<\rho<1,\sigma>0,t>0,\beta>0,\epsilon>0$, and set $k:=0$.
*Step* 1.: If $\Vert F(x_{k}) \Vert <\epsilon$, stop; otherwise go to Step 2.
*Step* 2.: Compute
  2.3 $$ d_{k}= \textstyle\begin{cases}-F(x_{k})& \text{if } k=0,\\ - ( 1+\beta_{k}\frac{F(x_{k})^{\top}d_{k-1}}{ \Vert F(x_{k}) \Vert ^{2}} )F(x_{k})+\beta_{k}d_{k-1} &\text{if } k\geq1, \end{cases} $$
  where $\beta_{k}$ is such that
  2.4 $$ \vert \beta_{k} \vert \leq t\frac{ \Vert F(x_{k}) \Vert }{ \Vert d_{k-1} \Vert },\quad \forall k\geq1. $$
*Step* 3.: Find the trial point $y_{k}=x_{k}+\alpha_{k} d_{k}$, where $\alpha_{k}=\beta\rho^{m_{k}}$ and $m_{k}$ is the smallest nonnegative integer *m* such that
  2.5 $$ -\bigl\langle F\bigl(x_{k}+\beta\rho^{m}d_{k} \bigr),d_{k}\bigr\rangle \geq\sigma\beta\rho^{m} \Vert d_{k} \Vert ^{2}. $$
*Step* 4.: Compute
  2.6 $$ x_{k+1}=P_{H_{k}}\bigl[x_{k}- \xi_{k}F(y_{k})\bigr], $$
  where
  $$H_{k}=\bigl\{ x\in R^{n}|h_{k}(x)\leq0\bigr\} , $$
  with
  2.7 $$ h_{k}(x)=\bigl\langle F(y_{k}),x-y_{k} \bigr\rangle , $$
  and
  $$\xi_{k}=\frac{\langle F(y_{k}), x_{k}-y_{k}\rangle}{ \Vert F(y_{k}) \Vert ^{2}}. $$
  Set $k:=k+1$ and go to Step 1.

Obviously, Algorithm 2.1 is different from the methods in [13, 14].

Now, we give some comment on the searching direction $d_{k}$ defined by (2.3). We claim that it is derived from Schmidt orthogonalization. In fact, in order to make $d_{k}=-F(x_{k})+\beta_{k}d_{k-1}$ satisfy the property

2.8 $$ F(x_{k})^{\top}d_{k}=- \bigl\Vert F(x_{k}) \bigr\Vert ^{2}, $$

we only need to ensure that $\beta_{k}d_{k-1}$ is vertical to $F(x_{k})$. As a matter of fact, by Schmidt orthogonalization, we have

$$d_{k}=- \biggl( 1+\beta_{k} \frac{F(x_{k})^{\top}d_{k-1}}{ \Vert F(x_{k}) \Vert ^{2}} \biggr)F(x_{k})+ \beta_{k}d_{k-1}. $$

Equality (2.8) together with the Cauchy-Schwarz inequality implies that $\Vert d_{k} \Vert \geq \Vert F(x_{k}) \Vert $. In addition, by (2.3) and (2.4), we have

$$\begin{aligned} \Vert d_{k} \Vert &\leq\bigl\Vert F(x_{k}) \bigr\Vert + \vert \beta_{k} \vert \frac{ \Vert F(x_{k}) \Vert \Vert d_{k-1} \Vert }{ \Vert F(x_{k}) \Vert ^{2}} \bigl\Vert F(x_{k}) \bigr\Vert + \vert \beta_{k} \vert \Vert d_{k-1} \Vert \\ & \leq\bigl\Vert F(x_{k}) \bigr\Vert +t \bigl\Vert F(x_{k}) \bigr\Vert +t \bigl\Vert F(x_{k}) \bigr\Vert \\ & =(1+2t) \bigl\Vert F(x_{k}) \bigr\Vert . \end{aligned}$$

Therefore, for all $k\geq0$, it holds that

2.9 $$ \bigl\Vert F(x_{k}) \bigr\Vert \leq \Vert d_{k} \Vert \leq(1+2t) \bigl\Vert F(x_{k}) \bigr\Vert . $$

Furthermore, it is easy to see that the line search (2.5) is well defined if $F(x_{k})\neq0$.

For parameter $\beta_{k}$ defined by (2.4), it has many choices such as $\beta_{k}^{\mathrm{S1}}= \Vert F(x_{k}) \Vert / \Vert d_{k-1} \Vert $, or [13, 14]

$$\begin{aligned} &\beta_{k}^{\mathrm{NWYL}}=\frac{\langle F(x_{k}), F(x_{k})-\frac{ \Vert F(x_{k}) \Vert }{ \Vert F(x_{k-1}) \Vert }F(x_{k-1})\rangle}{ \vert F(x_{k})^{\top}d_{k-1} \vert +t \Vert F(x_{k}) \Vert \Vert d_{k-1} \Vert }, \\ &\beta_{k}^{\mathrm{NPRP}}=\frac{\langle F(x_{k}), F(x_{k})-F(x_{k-1})\rangle}{\max\{t \Vert d_{k-1} \Vert , \Vert F(x_{k-1}) \Vert ^{2}\}}. \end{aligned}$$

From the structure of $H_{k}$, the orthogonal projection onto $H_{k}$ has a closed-form expression. That is,

$$P_{H_{k}}(x)= \textstyle\begin{cases}x-\frac{\langle F(y_{k}),x-y_{k}\rangle}{ \Vert F(y_{k}) \Vert ^{2}}F(y_{k})&\text{if } \langle F(y_{k}),x-y_{k}\rangle>0,\\ x&\text{if } \langle F(y_{k}),x-y_{k}\rangle\leq0. \end{cases} $$

### Lemma 2.1

*For function* $h_{k}(x)$ *defined by* (2.7), *let* $x^{*}\in S^{*}$, *it holds that*

2.10 $$ h_{k}(x_{k})\geq\sigma \Vert x_{k}-y_{k} \Vert ^{2} \quad\textit{and}\quad h_{k}\bigl(x^{*}\bigr)\leq0. $$

*In particular*, *if* $x_{k}\neq y_{k}$, *then* $h_{k}(x_{k})>0$.

### Proof

From $x_{k}-y_{k}=-\alpha_{k}d_{k}$ and the line search (2.5), we have

$$\begin{aligned} h_{k}(x_{k})&=\bigl\langle F(y_{k}),x_{k}-y_{k} \bigr\rangle \\ & =-\alpha_{k}\bigl\langle F(y_{k}),d_{k}\bigr\rangle \geq\alpha_{k}^{2}\sigma \Vert d_{k} \Vert ^{2}=\sigma \Vert x_{k}-y_{k} \Vert ^{2}. \end{aligned}$$

On the other hand, from condition (2.1), we can obtain

$$\begin{aligned} &h_{k}\bigl(x^{*}\bigr)=\bigl\langle F(y_{k}),x^{*}-y_{k} \bigr\rangle \leq0. \end{aligned}$$

This completes the proof. □

Lemma 2.1 indicates that the hyperplane $\partial H_{k}=\{x\in R^{n}|h_{k}(x)=0\}$ strictly separates the current iterate from the solutions of problem (1.1) if $x_{k}$ is not a solution. In addition, from Lemma 2.1, we also can derive that the solution set $S^{*}$ of problem (1.1) is included in $H_{k}$ for all *k*.

Certainly, if Algorithm 2.1 terminates at step *k*, then $x_{k}$ is a solution of problem (1.1). So, in the following analysis, we assume that Algorithm 2.1 always generates an infinite sequence $\{x_{k}\}$. Based on the lemma, we can establish the convergence of the algorithm.

### Theorem 2.1

*If* *F* *is continuous and condition* (2.1) *holds*, *then the sequence* $\{x_{k}\}$ *generated by Algorithm* 2.1 *globally converges to a solution of problem* (1.1).

### Proof

First, we show that the sequences $\{x_{k}\}$ and $\{y_{k}\}$ are both bounded. In fact, it follows from $x^{*}\in H_{k}$, (2.1), (2.2) and (2.6) that

$$\begin{aligned} \bigl\Vert x_{k+1}-x^{*} \bigr\Vert ^{2}&\leq \bigl\Vert x_{k}-\xi_{k}F(y_{k})-x^{*} \bigr\Vert ^{2} \\ &= \bigl\Vert x_{k}-x^{*} \bigr\Vert ^{2}-2 \xi_{k}\bigl\langle F(y_{k}),x_{k}-x^{*}\bigr\rangle +\xi_{k}^{2} \bigl\Vert F(y_{k}) \bigr\Vert ^{2} \\ &\leq \bigl\Vert x_{k}-x^{*} \bigr\Vert ^{2}-2 \xi_{k}\bigl\langle F(y_{k}),x_{k}-y_{k} \bigr\rangle +\xi_{k}^{2} \bigl\Vert F(y_{k}) \bigr\Vert ^{2} \\ &= \bigl\Vert x_{k}-x^{*} \bigr\Vert ^{2}- \frac{\langle F(y_{k}),x_{k}-y_{k}\rangle^{2}}{ \Vert F(y_{k}) \Vert ^{2}} \\ &\leq \bigl\Vert x_{k}-x^{*} \bigr\Vert ^{2}- \frac{\sigma^{2}\alpha_{k}^{4} \Vert d_{k} \Vert ^{2}}{ \Vert F(y_{k}) \Vert ^{4}}. \end{aligned}$$

Thus the sequence $\{ \Vert x_{k}-x^{*} \Vert \}$ is decreasing and convergent, and hence the sequence $\{x_{k}\}$ is bounded, and from (2.9), the sequence $\{d_{k}\}$ is also bounded. Then, by $y_{k}=x_{k}+\alpha_{k} d_{k}$, the sequence $\{y_{k}\}$ is also bounded. Then, by the continuity of $F(\cdot)$, there exists constant $M>0$ such that $\Vert F(y_{k}) \Vert \leq M$ for all *k*. So,

2.11 $$ \bigl\Vert x_{k+1}-x^{*} \bigr\Vert ^{2}\leq \bigl\Vert x_{k}-x^{*} \bigr\Vert ^{2}-\frac {\sigma^{2}\alpha_{k}^{4} \Vert d_{k} \Vert ^{4}}{M^{2}}, $$

from which we can deduce that

2.12 $$ \lim_{k\rightarrow\infty}\alpha_{k} \Vert d_{k} \Vert =0. $$

If $\liminf_{k\rightarrow\infty} \Vert d_{k} \Vert =0$, then from (2.9) it holds that $\liminf_{k\rightarrow\infty} \Vert F(x_{k}) \Vert =0$. From the boundedness of $\{x_{k}\}$ and the continuity of $F(\cdot)$, $\{x_{k}\}$ has some accumulation point *x̄* such that $F(\bar{x})=0$. Then from (2.11), $\{ \Vert x_{k}-\bar{x} \Vert \}$ converges, and thus the sequence $\{x_{k}\}$ globally converges to *x̄*.

If $\liminf_{k\rightarrow\infty} \Vert d_{k} \Vert >0$, from (2.9) again, we have

2.13 $$ \liminf_{k\rightarrow\infty} \bigl\Vert F(x_{k}) \bigr\Vert >0. $$

By (2.12), it holds that

2.14 $$ \lim_{k\rightarrow\infty}\alpha_{k}=0. $$

Therefore, from the line search (2.5), we have

2.15 $$ -\bigl\langle F\bigl(x_{k}+\beta\rho ^{m_{k}-1}d_{k}\bigr),d_{k}\bigr\rangle < \sigma\beta \rho^{m_{k}-1} \Vert d_{k} \Vert ^{2}. $$

Since $\{x_{k}\}$ and $\{d_{k}\}$ are both bounded, then letting $k\rightarrow\infty$ in (2.15) yields that

2.16 $$ -\bigl\langle F(\bar{x}),\bar{d}\bigr\rangle \leq0, $$

where $\bar{x},\bar{d}$ are limits of corresponding sequences. In addition, from (2.8) and (2.13), we get

2.17 $$ -\bigl\langle F(\bar{x}),\bar{d}\bigr\rangle = \bigl\Vert F( \bar{x}) \bigr\Vert ^{2}>0. $$

Obviously, (2.16) contradicts (2.17). This completes the proof. □

## Numerical results

In this section, numerical results are provided to substantiate the efficacy of the proposed method. The codes are written in Mablab R2010a and run on a personal computer with 2.0 GHz CPU processor. For comparison, we also give the numerical results of the spectral gradient method (denoted by SGM) in [9], the conjugate gradient method (denoted by CGM) in [16]. The parameters used in Algorithm 2.1 are set as $t=1,\sigma=0.01,\rho=0.5,\beta =1$, and

$$\beta_{k}=\frac{ \Vert F(x_{k}) \Vert }{ \Vert d_{k-1} \Vert }. $$

For SGM, we set $\beta=0.4,\sigma=0.01, r=0.001$. For CGM, we choose $\rho= 0.1, \sigma=10^{-4}$ and $\xi=1$. Furthermore, the stopping criterion is set as $\Vert F(x_{k}) \Vert \leq10^{-6}$ for all the tested methods.

### Problem 1

The mapping $F(\cdot)$ is taken as $F(x)=(f_{1}(x), f_{2}(x),\ldots,f_{n}(x))^{\top}$, where

$$f_{i}(x)=e^{x_{i}}-1 \quad\text{for } i=1,2,\ldots,n. $$

Obviously, this problem has a unique solution $x^{*}=(0,0,\ldots,0)^{\top}$.

### Problem 2

The mapping $F(\cdot)$ is taken as $F(x)=(f_{1}(x), f_{2}(x),\ldots,f_{n}(x))^{\top}$, where

$$\begin{aligned} &f_{1}(x)=x_{1}-e^{\cos(\frac{x_{1}+x_{2}}{n+1})}, \\ &f_{i}(x)=x_{i}-e^{\cos(\frac{x_{i-1}+x_{i}+x_{i+1}}{n+1})}, \quad\text{for } i=2,3, \ldots,n-1, \\ &f_{n}(x)=x_{n}-e^{\cos(\frac{x_{n-1}+x_{n}}{n+1})}. \end{aligned}$$

### Problem 3

The mapping $F(x): {R}^{4}\rightarrow{R}^{4}$ is given by

$$F(x)=\left ( \begin{matrix}1&0&0&0\\ 0&1&-1&0\\ 0&1&1&0\\ 0&0&0&0 \end{matrix} \right ) \left ( \begin{matrix}x_{1}\\ x_{2}\\ x_{3}\\ x_{4} \end{matrix} \right )+\left ( \begin{matrix}x_{1}^{3}\\ x_{2}^{3}\\ 2x_{3}^{3}\\ 2x_{4}^{3} \end{matrix} \right )+\left ( \begin{matrix}-10\\ 1\\ -3\\ 0 \end{matrix} \right ). $$

This problem has a degenerate solution $x^{*}=(2,0,1,0)$.

For Problem 1, the initial point is set as $x_{0}=(1,1,\ldots ,1)$, and Table 1 gives the numerical results by Algorithm 2.1 with different dimensions, where Iter. denotes the iteration number and CPU denotes the CPU time in seconds when the algorithm terminates. Table 2 lists the numerical results of Problem 2 with different initial points. The numerical results given in Table 1 and Table 2 show that the proposed method is efficient for solving the given two test problems.

**Table 1:** **Numerical results with different dimensions of Problem** **1**

**Dimension**	**10**	**50**	**100**	**500**	**1,000**
Iter.	20	21	22	23	23
CPU	0.3594	0.8750	1.7031	41.4844	257.1406

**Table 2:** **Numerical results with different initial points of Problem** **3**

**Initial point**	**Iter.**	**CPU**	$\boldsymbol {\Vert F(x_{k}) \Vert } $
(0,0,0,0)	20	0.9219	9.9320×10^−7^
(3,0,0,0)	22	0.8438	7.0942×10^−7^
(1,1,1,0)	41	1.6250	7.2649×10^−7^
(0,1,1,1)	28	1.1875	3.8145×10^−7^
(0,100,100,1)	34	1.4063	6.6320×10^−7^

## Conclusion

In this paper, we extended the conjugate gradient method to nonlinear equations. The major advantage of the method is that it does not need to compute the Jacobian matrix or any linear equations at each iteration, thus it is suitable to solve large-scale nonlinear constrained equations. Under mild conditions, the proposed method possesses global convergence.

In Step 4 of Algorithm 2.1, we have to compute a projection onto the intersection of the feasible set *C* and a half-space at each iteration, which is equivalent to quadratic programming, quite time-consuming work. Hence, how to remove this projection step is one of our future research topics.
